# DNA damage in peripheral blood mononuclear cells and neutrophils of dairy cows during the transition period

**Published:** 2012-06-25

**Authors:** M. Tharwat, D. Endoh, S. Oikawa

**Affiliations:** 1 *Department of Large Animal Clinical Sciences, School of Veterinary Medicine, Rakuno Gakuen University, 582 Bunkyodai-Midorimachi, Ebetsu, Hokkaido 069-8501, Japan*; 2 *Department of Animal Medicine, Faculty of Veterinary Medicine, Zagazig University, Egypt*; 3 *Department of Veterinary Radiology, School of Veterinary Medicine, Rakuno Gakuen University, 582 Bunkyodai-Midorimachi, Ebetsu, Hokkaido 069-8501, Japan*

**Keywords:** Apoptosis, Comet assay, Cow, Leukocytes, Transition period

## Abstract

This study was designed to investigate the apoptotic process in peripheral blood mononuclear cells (PBMC) and polymorphonuclear neutrophil leukocytes (PMN) in dairy cattle during the transition period. Blood samples were collected from 4 dairy cattle at 3 weeks before the expected parturition (wk -3), parturition (wk 0) and 3 weeks after parturition (wk +3). The DNA damage of PBMC and PMN was evaluated based on the comet assay using visual scoring (arbitrary units). Undamaged DNA remained within the core (score 0) and the broken DNA migrated from the core towards the anode forming the tail of a comet (scores 1-4). Significantly higher scores in PBMC at wk 0 and wk +3 were observed compared with those in PMN although there were no significant changes of scores in either cell type during the experimental period. It is suggested that the apoptotic rate of PBMC is accelerated compared with that of PMC during the transition period.

## Introduction

In dairy cattle, the importance of the transition period has been highlighted in several review articles (Mulligan and Doherty, 2008). This period is characterized by marked changes in the endocrine status of the animals that are much more dramatic than at any other time in the lactation gestation cycle, and by a reduction in feed intake when nutrient demand for the developing conceptus and the impending lactogenesis are increasing (Ingvartsen *et al.*, 2003). Transition cattle also experience immunosuppression in the periparturient period and often have to cope with sudden dietary changes that cause digestive disturbances.

In addition to the metabolic, endocrine, and immune system perturbations, transition dairy cattle are also likely to experience environmental stressors arising from the ordinary group changes that are associated with dairy farm management of dry and lactating cows (Mulligan and Doherty, 2008).

Leukocytes from dairy cows provide an excellent model for studying periparturient immune suppression, as these cells exhibit impaired inflammatory responses associated with leukocytosis and increased susceptibility of the animals to opportunistic bacteria such as gram-negative coliforms that cause mastitis (Kehrli and Harp, 2001). Specifically, neutrophil adhesion, migration, and phagocytosis-induced respiratory burst activities become depressed in some parturient cows (Burton and Erskine, 2003).

Recent studies have begun to elucidate potential molecular bases for certain parturition-induced neutrophil dysfunctions, showing that transcripts encoding key adhesion molecules, mitochondrial proteins, and ribosomal proteins are significantly decreased in neutrophils following the surge in blood steroids at parturition (Madsen *et al.*, 2002).

The single cell gel electrophoresis (SCGE) test, or comet assay, is a straightforward visual method for the detection of DNA damage in interphase cells. It is a direct, sensitive, simple, rapid and powerful technique used in toxicological studies. This technique is especially sensitive in detecting DNA single-strand breaks (SSBs), alkaline-labile damage and excision repair sites in individual cells. Compared with other classical methods of detection of DNA damage, SCGE has the advantage of showing DNA damage in individual cells.

The method can be used for small numbers of cells with relatively high sensitivity and provides a quantitative index for DNA damage (Mitchelmore and Chipman, 1998). The clear advantage of the comet assay over other techniques that measure DNA strand breaks is its ability to measure heterogeneity within complex populations. In the comet assay a damaged cell takes on the appearance of a comet, with head and tail regions.

It is generally assumed that SCGE performed under alkaline conditions primarily detects SSBs and alkali-labile sites in DNA (Tice *et al.*, 2000).

This study was designed to analyze the apoptotic process of peripheral blood mononuclear cells (PBMC) and polymorphonuclear neutrophil leukocytes (PMN) in dairy cows during the transition period.

## Materials and Methods

Details of this study design were performed as reported (Mohamed *et al.*, 2012). Briefly, four pregnant clinically healthy Holstein dairy cattle on a commercial farm in Hokkaido, Japan (milk yield of the herd, approximately 9,500 kg/y/cow) were selected based on physical examination and a complete blood test. Their age was 2.33±0.59 years, and all cows had body condition scores between 3.25 and 3.75 based on the 5-point scale of Edmonson *et al*. (1989).

These four cows had no clinical diseases during this study. All cattle were maintained in tie-stall barns following the *Laboratory Animal Control Guidelines* of Rakuno Gakuen University. Jugular blood sampling was carried out at 3 weeks before the expected parturition (wk -3), at parturition (wk 0, within 3 days from parturition) and 3 weeks after parturition (wk +3). Serum samples were stored at -40ºC until assay.

PBMC were isolated as described previously (Mohamed *et al.*, 2011). Briefly, 25 ml of phosphate-buffered saline (PBS) and 10 ml of Ficoll-Conray solution (specific gravity 1.078) were added to 10 ml of heparinized blood, which was then centrifuged at 1500 rpm at 25ºC for 30 min. The lymphocytes were isolated and washed 3 times in PBS, with centrifugation steps at 1500 rpm at 10ºC for 10 min. The cells were washed with PBS until the supernatant became clear. The resulting cell population comprised >95% lymphocytes, as determined by Wright-Giemsa staining, and < 99% of the cells were viable when assessed by trypan blue dye exclusion.

PMN were isolated from heparinized blood using Ficoll-Conray solution (specific gravity 1.078), followed by hypotonic red blood cell lysis, as we recently reported (Mohamed *et al.*, 2011). Neutrophils were added to Hanks’ balanced salt solution (HBSS, containing Ca^2+^ and Mg^2+^; Nissui Pharmaceutical Company, Japan) at a concentration of 5×10^6^ cells/ml. The resulting cell population comprised >95% neutrophils, as determined by Wright-Giemsa staining, and >99% of the cells were viable when assessed by trypan blue dye exclusion.

Apoptosis of PBMC and PMN was evaluated based on the comet assay under alkaline conditions (Mohamed *et al.*, 2011, 2012). In brief, PBMC and PMN were embedded in 1% low-melting-point agarose (Life Technologies Co., Ltd., Japan) and deposited on top of a 1% agarose base layer (Nakarai Techs Co., Ltd., Osaka, Japan) on fully frosted slides (Matsunami Glass Indust. Ltd., Tokyo, Japan).

After solidification of the top layer of agarose, the slides were placed in lysis buffer (2.5M NaCl, 100mM EDTA, 10mM Tris-HCl, 1% Na-sarcosinate, 10% dimethyl sulphoxide and 1% Triton X-100, pH 10.0) for one hour at 4ºC in a dark room. After lysis, the cell membranes and cytosol were removed while the isolated nuclei remained in the agarose. The slides were incubated in an electrophoretic buffer (0.3M NaOH, 1mM EDTA) for 30 min. Electrophoresis was carried out at 25 V and approximately 400 mA for 25 min at room temperature.

The slides were neutralized in 0.4M Tris-HCl solution (pH 7.5) for 20 min, stained with propidium iodide (PI), and then photographed under a fluorescent microscope (Olympus Optical Co., Ltd., Tokyo, Japan). Images were captured with a Sony CCD camera and saved using Image Pro Plus software. To evaluate DNA damage from comets, the comet visual scoring system was used (Collins, 2004). Generally, 100 comets were scored per slide, with each comet assigned a value of 0 to 4 according to its class. The total score for the sample gel then ranked form 0 to 400 in arbitrary units.

All statistical analyses were performed using computer software (SPSS version 17.0; SPSS, Chicago, Illinois, USA). The data were evaluated by repeated measures ANOVA. The significance of differences between the means in PBMC and PMN at each sampling time was analyzed by Student’s and Welch’s *t*-tests. Values were expressed as means ±SD.

## Results

**Fig. 1:**
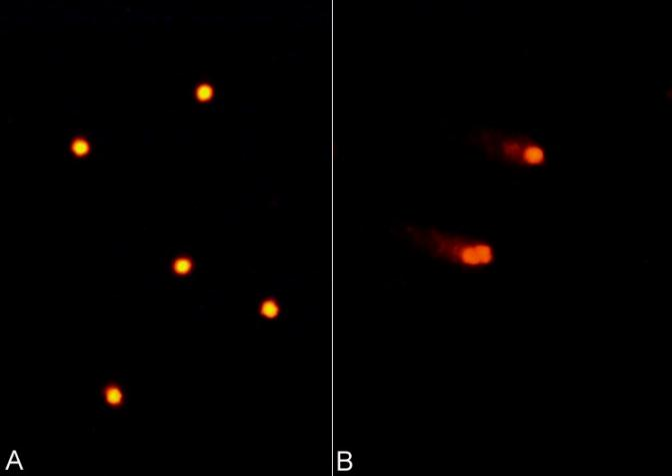
Comet images of peripheral blood mononuclear cells; **A:** In undamaged cells, the DNA is tightly compressed and maintains the circular disposition of the normal nucleus (wk −3, score 0). **B:** The damaged DNA migrates from the core toward the anode, forming the tail of a comet (wk +3, score 4).

In comet images of PBMC cells, undamaged DNA remained within the core (Figure 1A). The broken DNA, however, migrated from the core towards the anode, forming the tail of a comet (Figure 1B).

Fluorescenct microscopy revealed fluorescent structures corresponding to the PI–stained nuclear DNA of the PBMC. In undamaged cells, the DNA was tightly compressed and maintained the circular disposition of the normal nucleus.

**Fig. 2:**
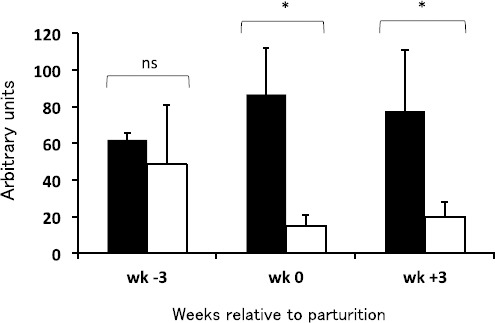
Arbitrary units of PBMC (black bars) and PMN (white bars) in dairy cows at 3 wk before parturition (wk −3), parturition (wk 0) and 3 wk postcalving (wk +3). *P < 0.05; ns, non-significant. There were no significant changes of the values in either cell type during the experimental period (three time points).

No significant changes of the values in PBMC occurred during the experimental period (Figure 2). Similar findings were also found for PMN. However, there were significant differences between the values for PBMC and PMN at calving (wk 0; P=0.012) and after parturition (wk +3; P=0.044).

## Discussion

During late pregnancy and parturition, mammals are subject to varying degrees of immunosuppression and disease susceptibility (Osterlundh *et. al*., 2001). Apoptosis of blood leukocytes has been evaluated using flow cytometric techniques (Sladek *et al.*, 2005). In this study, apoptosis of PBMC and PMN isolated from cows during the transition period was investigated and assessed based on the comet assay, using a visual scoring system (Collins, 2004).

This system has been reported to have very close agreement between the visual scoring method (arbitrary units) and sophisticated computer image analysis programs (percentage of DNA in the tail).

In this study, leukocyte apoptosis during the transition period may have been resulted from several factors. The increased circulating nonesterified fatty acid (NEFA) during the transition period may be an inducing factor. In our recent study, the elevated NEFA concentration during the transition period was associated with increased hepatocyte apoptosis (Mohamed *et al.*, 2012). Lipotoxicity towards leukocytes may also be an important factor. The proinflammatory and proapoptotic properties of several lipids (NEFA, free cholesterol and triglycerides) and lipid derivatives induce cellular dysfunction.

Long-term exposure to the lipids can induce lipotoxic injury, leading to excess secretion of proinflammatory cytokines and eventual cell death. Lipotoxicity affects vital cell functions (e.g., mitochondrial stress and membrane fluidity) through numerous pathways ending in the induction of cytokine release and finally apoptosis (Prieur *et al.*, 2010).

In bovines, it is well-established that the glucocorticoid level reaches its peak at parturition (Smith *et al.*, 1973). In humans, a glucocorticoid rise is reported to induce lymphocyte apoptosis (Planey and Litwack, 2000). In addition, glucocorticoid treatment of certain lymphoma cell lines and thymocytes activates a self-destructive pathway for programmed cell death of lymphocytes (Dowd *et al.*, 1991). On the other hand, several studies have reported that glucocorticoids attenuate apoptosis in human (Cox, 1995) and bovine neutrophils (Chang *et al.*, 2004). These may explain why, in the current study, the arbitrary units of PBMC were significantly higher than those of PMN at wk 0 and wk +3.

In conclusion, it is suggested that the apoptotic rate of PBMC is accelerated compared with that of PMN during the transition period.
